# Spatially Extended Relativistic Particles Out of Traveling Front Solutions of Sine-Gordon Equation in (1+2) Dimensions

**DOI:** 10.1371/journal.pone.0148993

**Published:** 2016-03-01

**Authors:** Yair Zarmi

**Affiliations:** The Jacob Blaustein Institutes for Desert Research, Ben-Gurion University of the Negev, Midreshet Ben-Gurion, 8499000, Israel; São Paulo State University, BRAZIL

## Abstract

Slower-than-light multi-front solutions of the Sine-Gordon in (1+2) dimensions, constructed through the Hirota algorithm, are mapped onto spatially localized structures, which emulate free, spatially extended, massive relativistic particles. A localized structure is an image of the junctions at which the fronts intersect. It propagates together with the multi-front solution at the velocity of the latter. The profile of the localized structure obeys the linear wave equation in (1+2) dimensions, to which a term that represents interaction with a slower-than-light, Sine-Gordon-multi-front solution has been added. This result can be also formulated in terms of a (1+2)-dimensional Lagrangian system, in which the Sine-Gordon and wave equations are coupled. Expanding the Euler-Lagrange equations in powers of the coupling constant, the zero-order part of the solution reproduces the (1+2)-dimensional Sine-Gordon fronts. The first-order part is the spatially localized structure. PACS: 02.30.Ik, 03.65.Pm, 05.45.Yv, 02.30.Ik.

## 1. Introduction

### 1.1 Motivation

#### 1.1.a Particles from travelling wave solutions

Soliton solutions of nonlinear evolution equations in (1+1) dimensions are localized in position at any time. Hence, in some studies of classical systems, such solitons have been viewed as emulation of spatially extended but localized particles with finite masses:

$$m=\int_{-\infty }^{+\infty }u\left(t,\;x\right)\;dx.$$ (1)

In Eq (1), *u*(*t*,*x*) is a single-soliton solution. Examples of this approach are the dynamics of solitons subjected to external forces [1, 2], the peakon (a localized structure) [3], the compacton, (a solitary wave solution of finite support) [4] and localized static solitons in KdV-type systems [5].

Localized solutions, for which

$$\int_{-\infty }^{+\infty }\left|u{\left(t,\;x\right)}^{2}\right|\;dx<\infty ,$$ (2)

have been viewed as candidates for normalizable wave functions, to be used as starting points for the construction of bound states within the framework of Quantum-Field Theoretic models. This idea has been discussed extensively in the High Energy literature (see, e.g., [6–16].) In particular, the connection between solutions of the (1+1)-dimensional Sine-Gordon equation and particles within a field-theoretic context has been studied (see, e.g., [14]).

There are nonlinear evolution-equations in more than one space dimension that have spatially localized solutions, which may be viewed as emulating spatially extended particles. Prime examples in the classical arena are the Kadomtsev-Petviashvili I equation [17, 18], the Davey-Stewartson equation [19, 20], the Gardner equation [21, 22] and the Nizhnik-Veselov-Novikov system [23–25]. The search for localized solutions in Quantum-Field Theory began with the discovery of the ‘t Hooft-Polyakov monopole [6, 7]—a spatially localized solution of the (1+3)-dimensional nonlinear Klein-Gordon equation.

However, there are higher-dimensional evolution equations, the solutions of which are not localized in space. For example, the travelling wave solutions of the (1+2)-dimensional Kadomtsev-Petviashvili II equation (KP II) are solitons, and those of the higher-dimensional Sine-Gordon equation are fronts. Still, localized structures, which emulate spatially extended particles, can be generated from such solutions in two or three space dimensions by a procedure that is a natural consequence of the evolution equation considered. Multi-soliton solutions of KP II were mapped onto localized structures, which emulate spatially extended non-relativistic particles that undergo collisions in the *x*-*y* plane [26]. The (1+2)-dimensional Sine-Gordon equation offers an interesting possibility. Its slower-than-light multi-front solutions can be mapped onto positive definite, spatially localized structures, which emulate free, spatially extended, massive relativistic particles.

The localized structures are generated by a functional, *R*[*u*], of a multi-wave solution, *u*. *R*[*u*] emerges naturally from the evolution equation in the cases of both the KP II equation [26], and of the Sine-Gordon equation. *R*[*u*] vanishes on the single-wave solution (soliton for KP II, front for Sine-Gordon), and maps multi-wave solutions onto structures that are localized around junctions, at which the waves intersect. In the case of the Sine-Gordon equation, *R*[*u*] obeys the linear wave equation, driven by a localized source term. This physical picture can be cast in the form of the Euler-Lagrange equations of a (1+2)-dimensional dynamical system, in which the Sine-Gordon and linear wave equations are weakly coupled.

#### 1.1.b Mechanism for mass generation

The generation or modification of the mass of a particle, or of an elementary excitation, appears many times in many areas of physics. The simplest manner to generate a mass, is to include a mass term in the dynamical equation of the field, as, for example, in the Klein-Gordon equation:

$$\partial_{\mu }\partial^{\mu }\rho \;+\;m_{0}{}^{2}\;\rho =0.$$ (3)

A more intricate manner for mass generation is the incorporation of interaction with an external field. The range of examples is enormous, from the generation of the effective mass of an electron in a crystal, to the generation of the mass of hadrons through the interaction with the Higgs Boson.

The system discussed here is akin to the generation of the effective mass of an electron in a solid, which is a consequence of the interaction of the electron with the ionic electromagnetic field. In the present case, a dynamical variable, *ρ*, obeys the (1+2)-dimensional linear wave equation, which is driven by an “external field” generated from a multi-front solution of the (1+2)-dimensional Sine-Gordon equation. The driving term is localized around the junction or junctions, at which fronts intersect. The driven wave equation admits for *ρ* a spatially localized, positive definite solution, which emulates a free, spatially extended, massive relativistic particle.

#### 1.1.c Mass as space integral of structure profile

In the interpretation of localized structures as spatially extended particles in nonlinear dynamics, the mass of a “particle” is usually defined as the space-integral of structure profile, as, e.g., in Eq (1). In the case of localized structures on the surface of an incompressible fluid, this choice makes sense, because the structure volume is proportional to the mass of the fluid it contains. (Well-known examples are soliton solutions of the KdV equation or of the Camassa-Holms peakons.) However, in general, there is no physical argument that requires this definition.

In the approach presented in Ref. [26] (KP II equation) and in this paper (Sine-Gordon equation in (1+2) dimensions), the definition of “particle” mass as the space integral of the profile of a localized structure bears fruits of physical significance. In the case of the non-relativistic “particles” generated from multi-soliton solutions of the KP II equation, this is the *only* definition of mass that ensures mass and linear momentum conservation in “particle” collisions. In the case of the (1+2)-dimensional Sine-Gordon equation discussed here, this definition allows for the interpretation of the localized structures as emulating free, spatially extended, massive relativistic particles.

#### 1.1.d Coupled nonlinear wave equations

There is a wealth of literature that deals with the solutions and the possible integrability of coupled Sine-Gordon or nonlinear Klein-Gordon equations in (1+1) dimensions (see, e.g., Refs. [27–36]). The generic form of such systems is:

$$w_{tt}\;-\;w_{xx}\;+\;f\left[w,\;\rho \right]=0\;,\;\rho_{tt}\;-\;\rho_{xx}\;+\;g\left[w,\;\rho \right]=0.$$ (4)

One case that has attracted great interest is the emergence of the Sine-Gordon equation in (1+1) dimensions in the description of the structure of DNA chains. (See, e.g., [37–57].) In particular, it has been proposed that the dynamics of a deformed DNA chain is governed by a perturbed Sine-Gordon equation coupled to a linear wave equation in (1+1) dimensions [55]. *ρ* then represents the longitudinal displacement from equilibrium of the position of a nucleotide in a chain.

The present paper presents an extension of such systems into (1+2) dimensions, with a specific form of the coupling of the Sine-Gordon equation and the linear wave equation:

$$w_{tt}\;-\;w_{xx}\;-\;w_{yy}\;+\text{ sin}w=g\;\rho \;F\left[w\right]\;,\;\rho_{tt}\;-\;\rho_{xx}\;-\;\rho_{yy}=g\;G\left[w\right].$$ (5)

For the forms assigned to the functionals *F*[*w*] and *G*[*w*] in this paper, and with appropriate boundary conditions, the system of Eq (5) admits a solution, which, through first order in the coupling constant, *g*, describes the (1+2)-dimensional front solutions of the Sine-Gordon equation, and a positive definite, localized solution of the driven wave equation.

### 1.2 Outline

The construction of solutions of the Sine-Gordon equation in (1+1) and (1+2) dimensions is reviewed in Section 2. The generation of localized structures from slower-than-light multi-front solutions is presented in Section 3. The interpretation of a localized structure as the mass density of a free, spatially extended relativistic particle and the source-driven wave equation obeyed by the structure are presented in Section 4. The Lagrangian formulation of the system of coupled Sine-Gordon and linear wave equations is presented in Section 5. The properties of tachyonic momentum vectors that are pertinent to the results presented here are discussed in Appendix I. Section 6 offers concluding comments.

## 2. Front Solutions of Sine-Gordon Equation

### 2.1 Review of (1+1) Dimensional Case

The Sine-Gordon equation [58–66],

$$\partial_{\mu }\;\partial^{\mu }u\;+\text{ sin}u=0,$$ (6)

is integrable in (1+1) dimensions [67]. The Hirota algorithm [68] for the construction of its travelling front solutions is based on a transformation:

$$u\left(x;\;Q\right)=4\;{\text{tan}}^{-1}\left[g\left(x;\;Q\right)/f\left(x;\;Q\right)\right].$$ (7)

(This transformation was first proposed in the special cases of one- and two-front solutions [64,65].) In Eq (7),

$$Q\equiv \left\{q^{\left(1\right)},\;q^{\left(2\right)},\;\ldots ,\;q^{\left(N\right)}\right\}.$$ (8)

*x* and *q* are, respectively, (1+1)-dimensional coordinate and momentum vectors, and

$$g\left(x;\;Q\right)=\sum_{\begin{array}{c} 1\leq n\leq N\\ n\;odd \end{array}}\left(\sum_{1\leq i_{1}<\cdots <i_{n}\leq N}\left\{\prod_{j=1}^{n}\varphi \left(x;q^{\left(i\right)}\right)\;\prod_{i_{l}<i_{m}}V\left(q^{\left(i_{l}\right)},\;q^{\left(i_{m}\right)}\right)\right\}\right),$$ (9)

$$f\left(x;\;Q\right)=1\;+\;\sum_{\begin{array}{c} 2\leq n\leq N\\ n\;even \end{array}}\left(\sum_{1\leq i_{1}<\cdots <i_{n}\leq N}\left\{\prod_{j=1}^{n}\varphi \left(x;q^{\left(i\right)}\right)\;\prod_{i_{l}<i_{m}}V\left(q^{\left(i_{l}\right)},\;q^{\left(i_{m}\right)}\right)\right\}\right),$$ (10)

$$\varphi \left(x;\;q^{\left(i\right)}\right)=e^{q_{\mu }{{}^{\left(i\right)}}_{\mu }\;x^{\mu }\;+\;\delta^{\left(i\right)}}.$$ (11)

In Eq (11), *δ*^(i)^ is a constant free phase. In addition, the momentum vectors are tachyonic,

$$q_{\mu }^{\left(i\right)}q^{\left(i\right)\;\mu }=-1,$$ (12)

and in Eqs (9) and (10),

$$V\left(q,\;q\prime \right)=\frac{1\;+\;q_{\mu }\;q\prime^{\mu }}{1\;-\;q_{\mu }\;q\prime^{\mu }}.$$ (13)

The fronts are mapped onto solitons in the current density:

$$J_{\mu }=\partial_{\mu }u\left(x\right).$$ (14)

Finally, *u*(*x*) is a Lorentz scalar; it is a function only of scalar products in Minkowski space (*x*·*q*^(i)^, *q*^(i)^·*q*^(j)^, 1 ≤ *i* ≠ *j* ≤ *N*). (This was first observed in the case of the single-front solution [65].) Given two reference frames that are connected by a Lorentz transformation, *L*, then:

$$u\left(x,\;\left\{q^{\left(1\right)},\;\ldots ,\;q^{\left(N\right)}\right\}\right)=u\left(\tilde{x},\;\left\{{\tilde{q}}^{\left(1\right)},\;\ldots ,\;{\tilde{q}}^{\left(N\right)}\right\}\right)\;\left(x=L\cdot \tilde{x},\;q^{\left(i\right)}=L\cdot {\tilde{q}}^{\left(i\right)},\;1\leq i\leq N\right).$$ (15)

### 2.2 Higher Space Dimensions

The attempt to extend the Hirota algorithm to the (1+2)-dimensional Sine-Gordon equation produced one- and two-front solutions, but encountered an obstacle in the case of three (let alone more than three) fronts [69]. For a three-front solution to exist, one of the three momentum vectors, from which the solution is constructed via Eqs (7)–(13), had to be a linear combination of the other two momenta. In later years, it was shown that the (1+2)-dimensional Sine-Gordon equation does not pass integrability tests employed in nonlinear dynamics [70–73].

The obstacle exposed by Hirota is the key to the extension of his algorithm to *N*-front solutions in (1+2) dimensions for any *N* ≥ 3 [74]. For a solution with *N* ≥ 3 fronts to exist, (*N*−2) of the momentum vectors in Eqs (7)–(13) must be linear combinations of just two of them:

$$q^{\left(i\right)}=\alpha_{i}\;q^{\left(1\right)}\;+\;\beta_{i}\;q^{\left(2\right)}\;\left(3\leq i\leq N\right).$$ (16)

Owing to Eq (16), each (1+2)-dimensional multi-front solution propagates rigidly in the *x*-*y* plane at a constant velocity, $\vec{v}$. The solutions are divided into two subsets: Solutions with $\left|\vec{v}\right|\geq c=1$, and solutions with $\left|\vec{v}\right|<c$. These characteristics are discussed in the following, with the aid of Appendix I. This paper focuses on the slower-than-light solutions.

The Hirota algorithm generates front solutions also for the (1+3)-dimensional Sine-Gordon equation. The one-and two-front solutions are spatially rotated (1+2)-dimensional solutions. For a solution with *N* ≥ 3 fronts to exist, every triplet of momentum vectors used in the construction the solution through Eqs (7)–(13) must obey the constraint [75]:

$${\left|\begin{array}{ccc} q_{1}^{\left(1\right)} & q_{2}^{\left(1\right)} & q_{2}^{\left(1\right)}\\ q_{1}^{\left(2\right)} & q_{2}^{\left(2\right)} & q_{2}^{\left(2\right)}\\ q_{1}^{\left(3\right)} & q_{2}^{\left(3\right)} & q_{2}^{\left(3\right)} \end{array}\right|}^{2}={\left|\begin{array}{ccc} q_{1}^{\left(1\right)} & q_{2}^{\left(1\right)} & q_{0}^{\left(1\right)}\\ q_{1}^{\left(2\right)} & q_{2}^{\left(2\right)} & q_{0}^{\left(2\right)}\\ q_{1}^{\left(3\right)} & q_{2}^{\left(3\right)} & q_{0}^{\left(3\right)} \end{array}\right|}^{2}\;+\;{\left|\begin{array}{ccc} q_{1}^{\left(1\right)} & q_{3}^{\left(1\right)} & q_{0}^{\left(1\right)}\\ q_{1}^{\left(2\right)} & q_{3}^{\left(2\right)} & q_{0}^{\left(2\right)}\\ q_{1}^{\left(3\right)} & q_{3}^{\left(3\right)} & q_{0}^{\left(3\right)} \end{array}\right|}^{2}\;+\;{\left|\begin{array}{ccc} q_{2}^{\left(1\right)} & q_{3}^{\left(1\right)} & q_{0}^{\left(1\right)}\\ q_{2}^{\left(2\right)} & q_{3}^{\left(2\right)} & q_{0}^{\left(2\right)}\\ q_{2}^{\left(3\right)} & q_{3}^{\left(3\right)} & q_{0}^{\left(3\right)} \end{array}\right|}^{2}.$$ (17)

Eq (17) offers a rich variety of solutions. These contain a subset, which is of physical significance, and of interest for this paper: Solutions with *N* ≥ 2 fronts in (1+3) dimensions that propagate rigidly at a constant velocity, $\left|\vec{v}\right|<c$. (All other multi-front solutions are not physical: They contain clusters of fronts that propagate rigidly at velocities that exceed *c* = 1.)

For *N* = 2, the slower-than light solution is a direct consequence of the discussion in Appendix I. For *N* ≥ 3, such solutions are constructed from momentum vectors, with Eq (16) obeyed by each triplet of vectors. Then, Eq (17) is obeyed trivially: For each triplet of the *N* vectors, all four determinants in Eq (17) vanish separately. As a result, all multi-front solutions in (1+3) dimensions, which propagate rigidly at a velocity $\left|\vec{v}\right|<c$, are obtained by applying three-dimensional rotations to such (1+2)-dimensional solutions. Therefore, *this paper focuses on slower-than-light* (1+2)-*dimensional front solutions*.

### 2.3 Construction of Slower-Than-Light Solutions in (1+2) Dimensions

All slower-than-light front solutions can be obtained by first constructing *static* (stationary, time independent) solutions through Eqs (7)–(13) [74]. The momentum vectors then have the form:

$${\tilde{q}}^{\left(i\right)}=\left\{0,\text{ cos}\psi^{\left(i\right)},\text{ sin}\psi^{\left(i\right)}\right\}\;,\;\left(1\leq i\leq N\right).$$ (18)

The Lorentz invariant coefficients of Eq (13) then obtain the form:

$$V\left({\tilde{q}}^{\left(i\right)},\;{\tilde{q}}^{\left(j\right)}\right)={\left(\text{tan}\left(\frac{\psi^{\left(i\right)}\;-\;\psi^{\left(j\right)}}{2}\right)\right)}^{2}>0.$$ (19)

Employing (1+2)-dimensional Lorentz transformations with all $\left|\vec{v}\right|<c$ boost velocities to both the position vector, *x*, and the momentum vectors in all static solutions yields all slower-than-light moving solutions. The moving solutions are Lorentz invariant. The velocity of a solution is just the velocity of the Lorentz boost.

These properties of slower-than-light solutions are direct consequences of the fact that the scalar product of any pair of momentum vectors, from which the solution is constructed, obeys:

$$\left|q^{\left(i\right)}\cdot q^{\left(j\right)}\right|<1\;\left(1\leq i\neq j\leq N\right).$$ (20)

(See Appendix I.) Eq (20) is obeyed by the momentum vectors of the form of Eq (18), from which a static solution is constructed. Hence, it is obeyed by the vectors after they had been Lorentz-transformed to any other frame, in which the solution becomes a moving one. Furthermore, in the static solution, the momentum vectors, ${\tilde{q}}^{\left(i\right)}$, of Eq (18) lie in a plane, so that only two of them can be linearly independent, the others being linear combinations of the two:

$${\tilde{q}}^{\left(i\right)}=\alpha_{i}\;{\tilde{q}}^{\left(1\right)}\;+\;\beta_{i}\;{\tilde{q}}^{\left(2\right)}.$$ (21)

The coefficients, *α*_*i*_ and *β*_*i*_ are constrained by Eq (12). This guarantees that the Hirota constraint, Eq (16), is also obeyed by the momentum vectors, from which the moving solution is constructed, as it is obtained by a Lorentz transformation applied to a static solution.

This procedure is invertible. The momentum vectors, from which a moving solution with *N* ≥ 2 fronts is constructed via Eqs (7)–(13), obey Eq (16). In addition, all momentum pairs obey Eq (20). As a result, the solution propagates rigidly at a velocity, $\left|\vec{v}\right|<c$, given by Eq (I.4). Hence, a Lorentz transformation to a rest frame, where the solution is static, exists.

In (1+2) dimensions, multi-front solutions, which propagate at velocities, $\left|\vec{v}\right|\geq c$, also exist [74]. They are constructed via Eqs (7)–(13) with momentum vectors, for which all pairs obey:

$$\left|q^{\left(i\right)}\cdot q^{\left(j\right)}\right|\geq 1\;\left(1\leq i\neq j\leq N\right).$$ (22)

### 3. Mapping (1+2)-Dimensional Multi-Front Solutions onto Localized Structures

The first step is the derivation of an identity that is obeyed by the single-front solution in all space dimensions. The *x*-dependence of a single-front solution is of the form

$$u\left(x;\;q\right)=h\left(\xi \right)\;\left(\xi =q\cdot x\;+\;\delta ,\;q_{\mu }\;q^{\mu }=-1\right).$$ (23)

In Eq (23), *δ* is the constant free phase of Eq (11). Using Eqs (23) and (12), the Sine-Gordon equation for a single-front solution in any space dimension becomes

−h′′ + sin h=0. (24)

The fact that *h* vanishes at either *ξ* → −∞ or *ξ* → +∞, yields a first integral:

$$-\frac{1}{2}\;{\left(h\prime \right)}^{2}\;+\;\left(1-\text{ cos }h\right)=0.$$ (25)

The relativistically invariant form of Eq (25) in any moving frame is:

$$R\left[u\right]=\frac{1}{2}\;\partial_{\mu }u\;\partial^{\mu }u\;+\;\left(1-\text{ cos}u\right)=0.$$ (26)

Eq (26) is obeyed by any single-front solution in any space dimension. (This can be verified by direct substitution of the single-front solution.) However, it is violated by all multi-front solutions. *R*[*u*] then maps the solution onto structures that are localized in the vicinity of front junctions in *u*. As an example, consider the two-front solution. Based on Eqs (7)–(13), one can rewrite the solution as a function of the arguments of the exponential in Eq (11) (*x* and *q*^(i)^ are now vectors in (1+2) dimensions):

$$\xi_{i}=q^{\left(i\right)}\cdot x\;+\;\delta^{\left(i\right)}\;\left(i=1,\;2\right).$$ (27)

Substituting Eqs (7)–(13) in Eq (26), one obtains:

$$R\left[u\right]=\frac{64\;e^{\xi_{1}\;+\;\xi_{2}}\;V\left(q^{\left(1\right)},\;q^{\left(2\right)}\right)}{\left(1\;+\;V\left(q^{\left(1\right)},\;q^{\left(2\right)}\right)\right)\;\left\{1\;+\text{​}e^{2\;\xi_{1}}\;+\;e^{2\;\xi_{2}}\;+\;e^{2\;\left(\xi_{1}\;+\;\xi_{2}\right)}\;{\left(V\left(q^{\left(1\right)},\;q^{\left(2\right)}\right)\right)}^{2}\;+\;2\;e^{\xi_{1}\;+\;\xi_{2}}\;\left(1\;+\;V\left(q^{\left(1\right)},\;q^{\left(2\right)}\right)\right)\right\}}.$$ (28)

The sign of *V*(*q*^(1)^,*q*^(2)^) of Eq (13) determines the properties of *R*[*u*]. In the case of slower-than-light multi-front solutions, thanks to Eq (20), *V*(*q*^(1)^,*q*^(2)^) > 0. Consequently, *R*[*u*] is positive definite when computed for these solutions. In addition, *R*[*u*] then falls off exponentially along each front line away from the front junction. For example, along front no. 1, *ξ*_1_ is of *O*(1), whereas, away from the junction, |*ξ*_2_| is large. *R*[*u*] then falls off exponentially as:

$$R\left[u\right]\underset{\left|\xi_{2}\right|\to \infty }{\to }\frac{64\;e^{\xi_{1}}\;V\left(q^{\left(1\right)},\;q^{\left(2\right)}\right)}{\left(1\;+\;V\left(q^{\left(1\right)},\;q^{\left(2\right)}\right)\right)\;\left\{1\;+\;e^{2\;\xi_{1}}\;{\left(V\left(q^{\left(1\right)},\;q^{\left(2\right)}\right)\right)}^{2}\right\}}\;e^{-\left|\xi_{2}\right|}\;+\;O\left(e^{-2\;\left|\xi_{2}\right|}\right).$$ (29)

Finally, *R*[*u*] has a maximum:

$$0<R\left[u\left(\xi_{1}=\xi_{2}=\frac{1}{2}\text{ log}V\left(q_{1},\;q_{2}\right)\right)\right]|=\left(16\;V\left(q^{\left(1\right)},\;q^{\left(2\right)}\right)/{\left(1\;+\;V\left(q^{\left(1\right)},\;q^{\left(2\right)}\right)\right)}^{2}\right)\leq 4.$$ (30)

**Fig 1:**
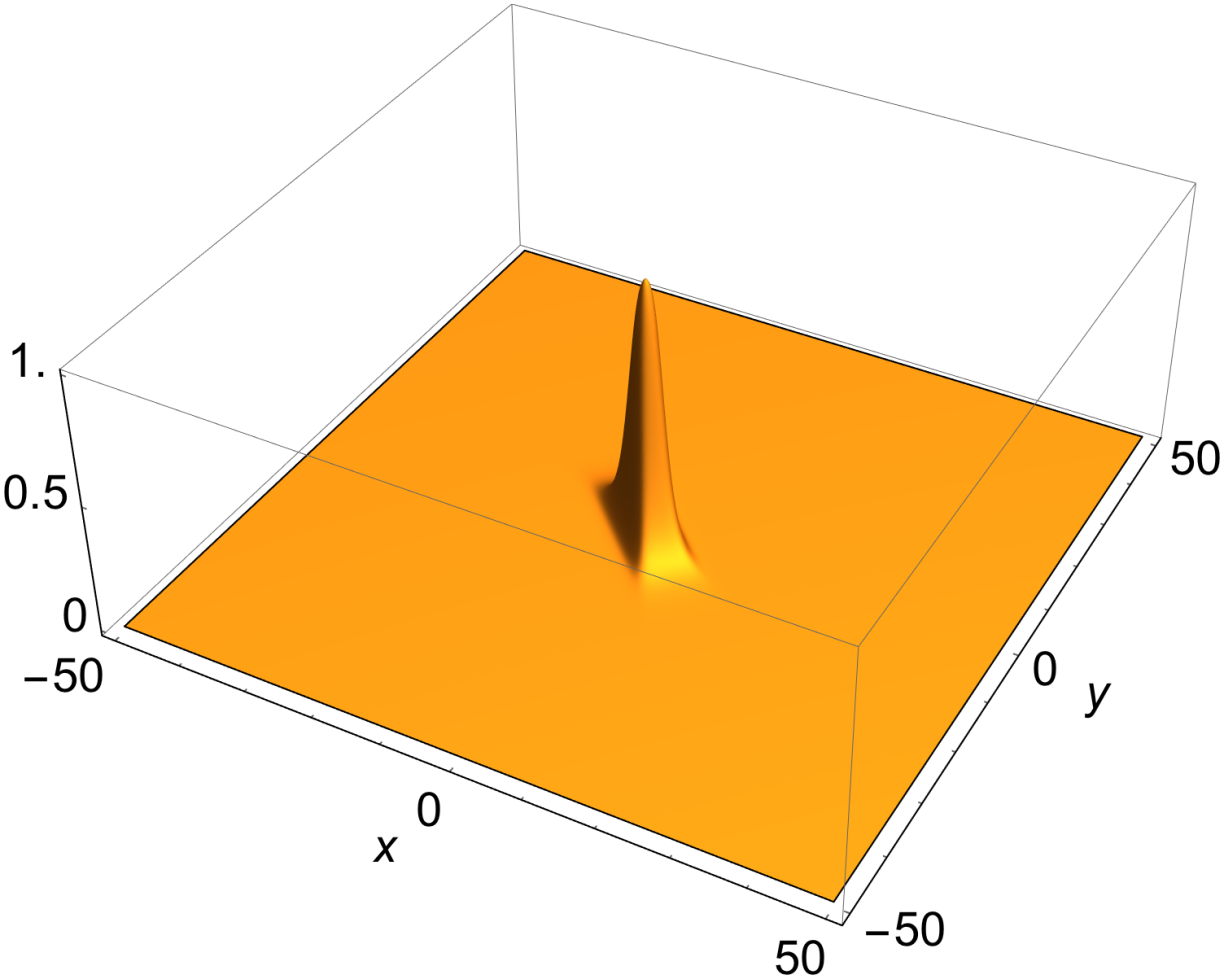
Vertex map of static two-front solution in (1+2) dimensions. Momenta (Eq (18)):*ψ*^(1)^ = *π*/6; *ψ* ^(2)^ = *π*/3; Phase shifts (Eq (11)): *δ*_1_ = *δ*_2_ = 0.

The image of a multi-front solution under *R*[*u*] will be called a *vertex map*. A vertex map of a slower-than-light multi-front solution moves in space at the velocity (*v* < *c*) of that solution. Like the solution, *u*, *R*[*u*] is also a Lorentz scalar. Moving solutions are obtained from static (1+2)-dimensional solutions by Lorentz transformations. Hence, it suffices to study static solutions. Fig 1 shows the vertex map of a static two-front solution. The case *V*(*q*^(1)^,*q*^(2)^) < 0 corresponds to front solutions that are faster-than-light, which are not discussed in this paper.

## 4. Particle Interpretation of Localized Structures

### 4.1 Source-Driven Wave Equation

The localized structure, *R*[*u*] of Eq (26), is *not* a solution of the Sine-Gordon equation. However, employing Eq (6) repeatedly, one obtains the following equation for *R*[*u*]:

$$\partial_{\mu }\partial^{\mu }R=\partial_{\mu }\partial_{\nu }u\;\partial^{\mu }\partial^{\nu }u\;-\;{\left(\partial_{\mu }\partial^{\mu }u\right)}^{2}.$$ (31)

With the aid of Eq (12), one finds that the driving term on the r.h.s. of Eq (31) vanishes identically when *u* is a single-front solution, given by Eq (23). Denoting *R* and *u* in the rest frame, where *u* is a static solution by, respectively *R*^(S)^ and *u*^(S)^, Eq (31) reduces in the rest frame to:

$$-\partial_{x}{}^{2}R\left[u^{\left(S\right)}\right]-\partial_{y}{}^{2}R\left[u^{\left(S\right)}\right]=2\;\left({\left(u_{xy}^{\left(S\right)}\right)}^{2}\;-u_{xx}^{\left(S\right)}\;u_{yy}^{\left(S\right)}\right).$$ (32)

The relativistically invariant driving term on the r.h.s. of Eq (31) may be expressed in another instructive form. One exploits the fact that all the momentum vectors, from which a multi-front solution is constructed, obey Eq (16). Hence, a multi-front solution with *N* ≥ 2 fronts depends only on two Lorentz scalars (together with the many *α*- and *β*-coefficients):

$$\xi_{i}=q_{\mu }^{\left(i\right)}\;x^{\mu }\;\left(i=1,\;2\right).$$ (33)

Repeated application of Eqs (6) and (12) to *R*[*u*] of Eq (26) converts Eq (31) into:

$$\partial_{\mu }\partial^{\mu }R\left[u\right]=2\;\left(1\;-\;{\left(q^{\left(1\right)}\cdot q^{\left(2\right)}\right)}^{2}\right)\;\left({\left(u_{\xi_{1}\;\xi_{2}}\right)}^{2}\;-\;u_{\xi_{1}\;\xi_{1}}\;u_{\xi_{2}\;\xi_{2}}\right).$$ (34)

**Fig 2:**
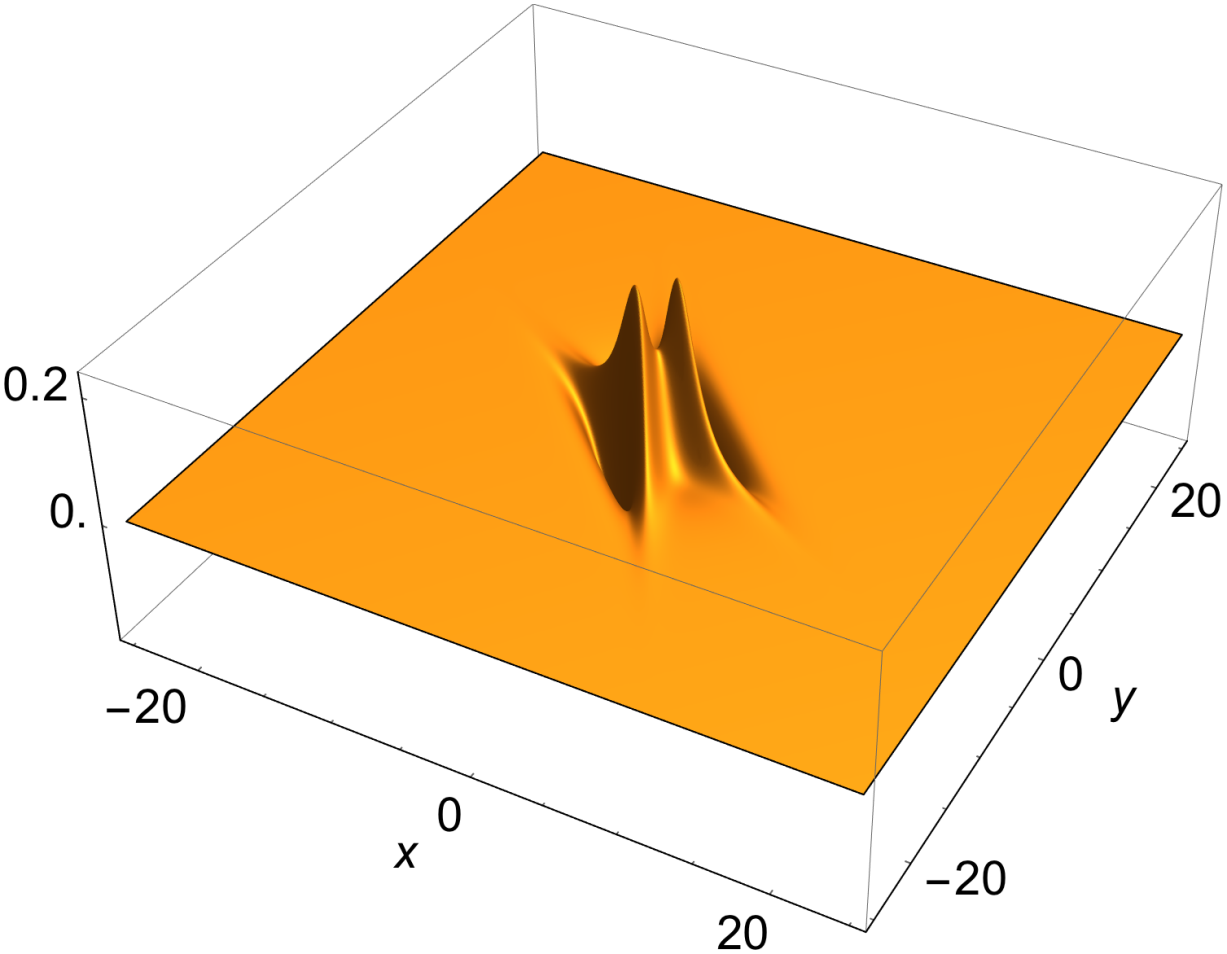
Source term in Eq (32), constructed from static two-front solution. Parameters as in Fig 1.

The driving term is localized around front junctions, but need not be positive definite. It vanishes identically on a single-front solution, obtained when all momenta become identical; both multiplicative factors on the r.h.s. of Eq (34) then vanish. An example of the driving term in Eq (32), generated from a two-front solution in its rest frame, is shown in Fig 2.

### 4.2 Definition of Mass

*R*[*u*] of Eq (26) looks similar to the Lagrangian- or the Hamiltonian-densities of the Sine-Gordon equation, but coincides with neither. The fact that *R*[*u*] is positive definite and spatially localized suggests that one interprets it as a mass density of a spatially extended particle. In relativistic physics, the wave equation is associated with massless particles. Often, a mass term is added, so as to generate solutions that correspond to massive particles, as, e.g., in the Klein-Gordon equation,

$$\partial_{\mu }\partial^{\mu }\rho +m_{0}{}^{2}\;\rho =0.$$ (35)

In the present case, mass is generated by the front-driven term on the r.h.s. of Eq (31).

To complete the particle analogy, one has to assign the structure a mass. With the proposed interpretation, the mass is given as the space integral of *R*[*u*(*x*)]:

$$m=\int R\left[u\left(x\right)\right]\;d^{2}\vec{x}.$$ (36)

A static solution is at rest; so is its vertex map, *R*[*u*]. Let us denote the rest mass of a vertex map by *m*_0_. The Hirota solutions of Eq (6) and, hence, *R*[*u*(*x*)] are Lorentz scalars. As a result, the mass of a moving “particle” differs from *m*_0_ only by the Jacobian of the space part of the Lorentz transformation that connects the moving and rest frames. This Jacobian is the Lorentz factor, *γ*:

$$m=m_{0}\;\gamma \;\left(\gamma =\left(1/\sqrt{1\;-\;v^{2}}\right)\right).$$ (37)

Thus, *R*[*u*] emulates the mass density of a free, spatially extended, massive relativistic article.

### 4.3 Constancy of Mass

Formally, the mass defined in Eq (36) need not be constant in time. From Eq (26), one obtains:

$$\frac{d}{dt}m=\int \partial_{t}R\left[u\left(x\right)\right]\;d^{2}\vec{x}=\int \left\{\partial_{\mu }u\;\partial^{\mu }u_{t}\;-\;u_{t}\;\partial_{\mu }\partial^{\mu }u\right\}\;d^{2}\vec{x},$$ (38)

which, for a general solution of Eq (6), has no reason to vanish. However a slower-than-light multi-front solution of Eq (6) in (1+2) dimensions, is obtained from the static solution by a Lorentz transformation. In the static solution, the “rest mass”, *m*_0_, is a number. As the moving solution is obtained from the static one through a Lorentz transformation, it propagates rigidly at the constant Lorentz boost velocity, and obeys Eq (I.8). Hence, the mass remains constant, the only change being the effect of Lorentz contraction, as in Eq (37).

This statement can be demonstrated more formally. As pointed out in Section 4.1, the *x*-dependence of an *N*-front solution is expressible in terms of two Lorentz scalars, *ξ*_1_ and *ξ*_2_ (see Eq (34) and the discussion preceding it). Consequently, so is the *x*-dependence of *R*[*u*]. (The case of a two-front solution, see Eq (28), provides an example.) Eq (36) can be re-expressed as:

m=|qx(1) qy(2) − qx(2) qy(1)| (∫R[u(x)] dξ1 dξ2)=γ 1 − (q(1)⋅q(2))2 (∫R[u(x)] dξ1 dξ2). (39)

The multiplicative factor in Eq (39) is the Jacobian for the transformation {*x*,*y*} → {*ξ*_1_,*ξ*_2_}. Thanks to Eq (I.5), it is proportional to the Lorentz factor, *γ*, thereby ensuring Eq (37).

### 4.4 “Bound States”?

**Fig 3:**
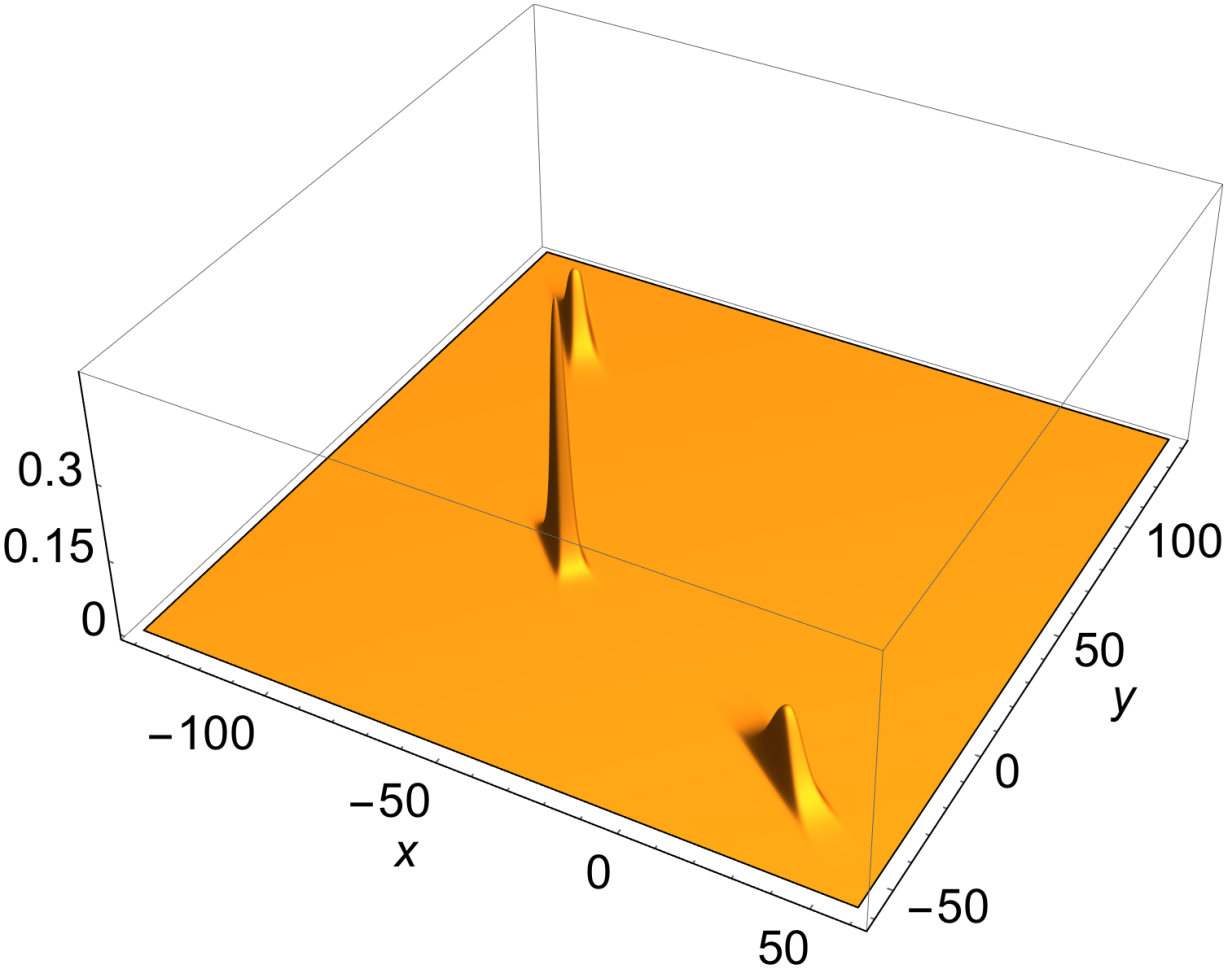
Vertex map of static three-front solution in (1+2) dimensions with phase-shifted fronts. Momenta (Eq (18)): *ψ* ^(1)^ = *π*/6; *ψ* ^(2)^ = *π*/3; *ψ* ^(3)^ = *π*/4; Phase shifts (Eq (11)): *δ*_1_ = −40; *δ*_2_ = −10; *δ*(3) = 0.

An interesting observation emerges in solutions with *N* ≥ 3 fronts. When all the free phases in Eq (11) are sufficiently small, there is only one front junction. In a static solution, it is localized around the origin in the *x*-*y* plane. If the phases are sufficiently sizable, then up to *N*(*N*−1)/2 distinct junctions (the maximal number of intersection points of *N* lines in the plane) may exist. *R*[*u*] of Eq (26) then generates up to *N*(*N*−1)/2 distinct, localized structures. Fig 3 shows a vertex map of a static 3-front solution, with constant free phases so chosen that there are three distinct vertices. In a moving frame, all three move rigidly together at the same velocity, preserving their profiles. *R*[*u*], comprised of the triplet of vertices, is a Lorentz scalar, and its mass obeys Eq (37). Each of the three vertices does not enjoy these properties. When they are close to one another, they are distorted. When far apart, each obeys Eq (39) approximately, as they are connected to one another by small exponential tails. Thus, the triplet emulates a free, spatially extended particle. This is suggestive of a primitive emulation of a “bound state”.

## 5. Lagrangian System

The coupling between the Sine-Gordon and wave equations in (1+2) dimensions, leading to Eq (31), can be obtained from the Euler-Lagrange equations of a system of two dynamical variables described by the following Lagrangian density:

$$\begin{array}{c} L=\frac{1}{2}\;\partial_{\mu }w\;\partial^{\mu }w\;-\;\left(1\;-\text{ cos}w\right)\;+\;\frac{1}{2}\;\partial_{\mu }\rho \;\partial^{\mu }\rho \;+\;g\;\rho \;J\left[w\right]\\ \left(\left|g\right|«1,\;\mu =0,\;1,\;2\right) \end{array}.$$ (40)

*L* is the sum of the Lagrangian densities of the Sine-Gordon and the linear wave equations, and a small coupling term. The Euler-Lagrange equations are:

$$\partial_{\mu }\partial^{\mu }w\;+\text{ sin}w=g\;\rho \;\frac{\delta J\left[w\right]}{\delta w},$$ (41)

$$\partial_{\mu }\partial^{\mu }\rho =g\;J\left[w\right].$$ (42)

Here *δ* denotes a variational derivative. Let us expand *w* and *ρ* in powers of *g*:

$$w=u\;+\;g\;u^{\left(1\right)}\;+\;g^{2}\;u^{\left(2\right)}\;+\;\ldots ,$$ (43)

$$\rho =\rho^{\left(0\right)}\;+\;g\;\rho^{\left(1\right)}\;+\;g^{2}\;\rho^{\left(2\right)}\ldots .$$ (44)

Inserting Eqs (43) and (44) in Eqs (41) and (42), the order-by-order equations through *O*(*g*) are:

$$\partial_{\mu }\partial^{\mu }u\;+\text{ sin}u=0,$$ (45)

$$\partial_{\mu }\partial^{\mu }\rho^{\left(0\right)}=0,$$ (46)

$$\partial_{\mu }\partial^{\mu }u^{\left(1\right)}\;+\;u^{\left(1\right)}\text{ cos}u=\rho^{\left(0\right)}\;\frac{\delta J\left[u\right]}{\delta u},$$ (47)

$$\partial_{\mu }\partial^{\mu }\rho^{\left(1\right)}=J\left[u\right].$$ (48)

Eq (45) is the (1+2)-dimensional Sine-Gordon equation, and Eq (46) is the linear wave equation, which has the capacity to generate massless particles (photons).

These dynamical equations may be exploited for the study of the effect of the interaction with Sine-Gordon fronts on the massless particle solutions of the zero-order equation, Eq (46). However, the results presented in Sections 3 and 4 indicate that the first-order Equation, Eq (48), naturally generates a, slower-than-light, localized solution. Focusing on the latter type of solutions, suggests another interesting research direction: One precludes the possibility of massless solutions, for which there is no rest frame, by imposing vanishing boundary conditions at infinity and by requiring that *ρ* is of *O*(*g*):

$$\rho^{\left(0\right)}=0.$$ (49)

Eq (47) is then homogeneous (its r.h.s. vanishes), allowing, in the same spirit, for imposing

$$u^{\left(1\right)}=0.$$ (50)

For solutions obeying Eqs (49) and (50), the *O*(*g*^2^) equations are found to be:

$$\partial_{\mu }\partial^{\mu }u^{\left(2\right)}\;+\;u^{\left(2\right)}\text{ cos}u=\rho^{\left(1\right)}\;\frac{\delta J\left[u\right]}{\delta u}$$ (51)

and

$$\partial_{\mu }\partial^{\mu }\rho^{\left(2\right)}=0.$$ (52)

To preclude the possibility of massless solutions, let us again choose

$$\rho^{\left(2\right)}=0.$$ (53)

In summary, with appropriate boundary conditions, the order-by-order expansion of the Euler-Lagrange equations of the system described by the Lagrangian of Eq (40) allows for a solution, which, in *O*(*g*^0^), is a front solution of the (1+2)-dimensional Sine-Gordon equation, and, the *O*(*g*) part is a solution of Eq (48), the linear wave equation, driven by a source-term that is constructed out of front solutions of Eq (45). Eq (50) for *ρ*^(1)^ coincides with Eq (31) for *R*[*u*] if one Chooses the source term, *J*[*w*] to coincide in form with the driving term in Eq (31),

$$J\left[w\right]=\partial_{\mu }\partial_{\nu }w\;\partial^{\mu }\partial^{\nu }w\;-\;{\left(\partial_{\mu }\partial^{\mu }w\right)}^{2},$$ (54)

Thus, the dynamical system described by the Lagrangian of Eq (40) allows for a solution, in which the massive, spatially extended particle-like structure affects Sine-Gordon fronts only in *O*(*g*^2^), and the Sine-Gordon fronts affect the spatially localized structure only in *O*(*g*^3^). A detailed analysis shows that these effects are also localized around front junctions.

### 6. Concluding Comments

1. The ideas presented in this paper may be summarized in terms of a system of two coupled equations, for two dynamical variables, one yielding the Sine-Gordon fronts, the other—a localized entity, which may be interpreted as the mass density of a free, spatially extended relativistic particle.
2. The resulting equations can be described in terms of a (1+2)-dimensional dynamical system that involves two degrees of freedom, with a Lagrangian density, in which the Sine-Gordon equation and the linear wave equation are coupled by a weak-coupling term.
3. Mass generation is through the interaction of the *ρ*-field in Eq (36) with an external field, *J*[*w*], generated (in lowest order) from (1+2)-dimensional Sine-Gordon fronts. This is akin to the generation of the effective mass of an electron in a crystal.
4. The ideas presented here may be applicable to other relativistically invariant evolution equations, which have travelling multi-wave (solitons or fronts) solutions. A single-wave identity, the analog of the vanishing of *R*[*u*] of Eq (26) on the single-Sine-Gordon front, needs to be derived. The resulting functional generates structures that are localized around wave junctions when computed for multi-wave solutions. These structures emulate spatially extended particles.
5. In the interpretation of localized structures as spatially extended particles, particle mass is often defined as the space-integral of structure profile. There is no physical argument that requires this definition, except in the case of localized structures on the surface of an incompressible fluid. In the approach presented in this paper (Sine-Gordon equation in (1+2) dimensions) and in Ref. [26] (KP II equation), the definition of “particle” mass as a space integral of the profile of a localized structure bears fruits of physical significance. In the case the KP II equation, this definition is the *only* one that ensures mass and linear momentum conservation in the non-relativistic “particle” collisions [26]. In the case of the (1+2)-dimensional Sine-Gordon equation, the definition of Eq (38) ensures that the localized structures emulate free, spatially extended, relativistic particles.

## Appendix I. Lorentz Transformations of Tachyonic (1+2)-Dimensional Momentum Vectors

A Lorentz transformation in (1+2) dimensions is given by the following matrix:

$$L=\left(\begin{array}{ccc} \gamma & -\gamma \;\beta_{x} & -\gamma \;\beta_{y}\\ -\gamma \;\beta_{x} & 1\;+\;\left(\gamma \;-\;1\right)\;\frac{\beta_{x}{}^{2}}{\beta^{2}} & \left(\gamma \;-\;1\right)\;\frac{\beta_{x}\;\beta_{y}}{\beta^{2}}\\ -\gamma \;\beta_{y} & \left(\gamma \;-\;1\right)\;\frac{\beta_{x}\;\beta_{y}}{\beta^{2}} & 1\;+\;\left(\gamma \;-\;1\right)\;\frac{\beta_{y}{}^{2}}{\beta^{2}} \end{array}\right).$$ (I.1)

In Eq (I.1), with *c* = 1,

$$\vec{\beta }=\left\{\beta_{x},\;\beta_{y}\right\}=\left\{v_{x},\;v_{y}\right\}\;,\;\gamma =1/\sqrt{1\;-\;{\vec{\beta }}^{2}},$$ (I.2)

where *v*_*x*_ and *v*_*y*_ are the components of the velocity of the Lorentz transformation.

A single-front solution is constructed in terms of one momentum vector. In solutions with *N* ≥ 3, only two of the momentum vectors are independent (see Eq (16)). Hence, it suffices to discuss the properties of one and two tachyonic momentum vectors under Lorentz transformations.

As there are two free parameters, *β*_*x*_ and *β*_*y*_, a single (1+2)-dimensional vector that obeys Eq (12), can always be transformed into the form of Eq (18) by a one-parameter family of transformations.

The situation is different when two vectors *q*^(1)^ and *q*^(2)^, which obey Eq (12), are considered. Applying the transformation of Eq (I.1) to the two vectors

$$q^{\left(i\right)}=\left\{q_{0}^{\left(i\right)},\;q_{x}^{\left(i\right)},\;q_{y}^{\left(i\right)}\right\}\;\left(i=1,\;2\right),$$ (I.3)

one obtains the expressions for *β*_*x*_ and *β*_*y*_, which are required for the transformed vectors to have vanishing time components, as in Eq (18):

$$\beta_{x}=-\frac{q_{0}^{\left(1\right)}\;q_{y}^{\left(2\right)}\;-\;q_{0}^{\left(2\right)}\;q_{y}^{\left(1\right)}}{q_{x}^{\left(1\right)}\;q_{y}^{\left(2\right)}\;-\;q_{x}^{\left(2\right)}\;q_{y}^{\left(1\right)}}\;,\;\beta_{y}=\frac{q_{0}^{\left(1\right)}\;q_{x}^{\left(2\right)}\;-\;q_{0}^{\left(2\right)}\;q_{x}^{\left(1\right)}}{q_{x}^{\left(1\right)}\;q_{y}^{\left(2\right)}\;-\;q_{x}^{\left(2\right)}\;q_{y}^{\left(1\right)}}.$$ (I.4)

For the transformation to be feasible, its velocity must be lower than *c* = 1. Hence, the magnitude of the vector $\vec{\beta }$ must be smaller than 1. Using Eqs (I.4) and (12), one obtains the constraint:

$$1\;-\;\beta_{x}{}^{2}\;-\;\beta_{y}{}^{2}=\frac{1\;-\;{\left(q^{\left(1\right)}\cdot q^{\left(2\right)}\right)}^{2}}{{\left(q_{x}^{\left(1\right)}\;q_{y}^{\left(2\right)}\;-\;q_{x}^{\left(2\right)}\;q_{y}^{\left(1\right)}\right)}^{2}}>0.$$ (I.5)

Thus, for a pair of vectors that obey Eq (12) to be Lorentz-transformable to the form given in Eq (18), its scalar product in Minkowski space must obey

$$\left|q^{\left(1\right)}\cdot q^{\left(2\right)}\right|<1.$$ (I.6)

With two momentum vectors given by Eq (18), Eqs (7)–(13) generate a static two-front solution of the (1+2)-dimensional Sine-Gordon equation. These vectors obey Eq (I.6). Lorentz transforming to a moving frame, Eq (I.6) is obeyed by the transformed vectors, and the pair of fronts moves rigidly at the velocity, *v* < *c*, given in Eq (I.4). If a solution contains *N* ≥ 3 fronts, then Eqs (16) and (I.6) guarantee that it also propagates rigidly as a whole at the same velocity.

Using $\vec{\beta }$, defined in Eqs (I.4) and (18) for the momentum vectors in the reference frame, at which a solution is static, one obtains the following identity for scalar products:

$$q^{\left(i\right)}\cdot x=-{\vec{q}}^{\left(i\right)}.\left(\vec{x}\;-\;\vec{\beta }\;t\right).$$ (I.7)

Thanks to Eq (I.7), a *v* < *c* solution with *N* ≥2 fronts, obeys (for the sake of clarity, the time and space parts of the position vector, *x*, are displayed explicitly):

$$u\left(t,\;\vec{x};\;Q\right)=u\left(0,\;\vec{x}\;-\;\vec{\beta }\;t;\;Q\right).$$ (I.8)

If the inequality (I.6) is inverted,

$$\left|q^{\left(1\right)}\cdot q^{\left(2\right)}\right|\geq 1,$$ (I.9)

then there is no velocity lower than *c* that can yield the desired transformation. The multi-front solution generated from such vectors propagates at a velocity, *v* ≥ *c* = 1.

Finally, the limit of equality,

$$q^{\left(1\right)}\cdot q^{\left(2\right)}=\pm 1,$$ (I.10)

cannot be reached from within the family of *v* < *c* solutions. A *v* < *c* solution with *N*-fronts degenerates into a *v* < *c* solution with (*N*−1) fronts in the case of the (+) sign, and (*N*−2) fronts in the case of the (−) sign. Hence, one cannot reach the speed of light from below. However, the limit of Eq (I.10) can be achieved within the family of *v* > *c* solutions, which obey Eq (I.9). The two solution subsets are not connected by a continuous operation.
